# “Zero to Hero”: Conceptualising Time as a Moderator of Nurses' Emotional Labour on the Front Line

**DOI:** 10.1155/2023/9383167

**Published:** 2023-08-18

**Authors:** Kate Kirk, Laurie Cohen, Stephen Timmons, Alison Edgley

**Affiliations:** ^1^Social Science Applied Healthcare and Improvement Research, University of Leicester, Leicester, UK; ^2^Nottingham University Business School, Nottingham, UK; ^3^School of Health Sciences, University of Nottingham, Nottingham, UK

## Abstract

**Aim:**

We aimed to conceptualise how environmental, institutional, and organisational dynamics of the ED underpin and “moderate” nurses' emotional labour.

**Background:**

Around the globe, EDs are struggling to meet rising patient demand including both UK health systems and public services in the US. In spite of these challenges and the intense and distinctive nature of EDs, an exploration of emotional labour is largely missing from current understanding. This is important, in part because emotional labour is established as an indicator of wellbeing including intention to leave, burnout, and compassion fatigue. We understand little of how the environment moderates emotional labour, and our study addressed this problem in the ED. Understanding the moderators of emotional labour, organisational perspective also offers theoretical development.

**Methods:**

Ethnography enabled immersion in the ED setting, gathering the lived experiences and narratives of the ED nursing team. This included 200 hours of observation at one District General Hospital and one Major Trauma Centre in the English NHS with 35 semistructured concurrent formal interviews.

**Results:**

/

**Conclusions:**

The ED calls for an extensive spectrum of emotional labour from staff, influenced and moderated by the restrictions on resources, particularly time. We argue that, despite the often short nature of interactions undertaken in ED, the labour required is effortful and gruelling for staff. Understanding the relevance of environmental elements, namely, time, to the emotional labour offers tangible opportunities for improvement. These new understandings can underpin solutions to negative consequences of this work. Suggested measures and interventions to alleviate the impact of emotional labour should be prioritised by policy makers and those tasked with managing, designing, and leading the delivery of care in ED. *Implications for Profession and/or Patient Care*. The more “sped up” a service is required to be, the higher the likelihood of emotional labour is. In light of the challenges facing healthcare services around the world and the increased throughput through services, particularly in ED, this is important. This is also critical when considering that there are well-established relationships between emotional labour and wellbeing in nursing. Understanding the relevance of the healthcare environment to staff members' experiences of emotional labour is critical in designing solutions which counterbalance the potentially negative consequences of this work.

## 1. Background

### 1.1. Pressure in Emergency Care

Internationally, demand for public health services is outstripping supply. There is an increased need for efficiency and quicker throughput through the services, while still meeting the expectations of patients and their relatives [1]. Both anecdotally and within academic literature, the emergency department (ED) has been described as a “window” into the broader healthcare organisation and as a representation of the national and international healthcare challenges facing communities and populations [2]. Many of the demands facing EDs [3] are as a direct consequence of, or are entwined with, broader societal pressures.

At the time of writing, time-critical “service indicators” in ED in the English National Health Service (NHS) used as indicators of “quality” have been at an all-time low with less than 60% of patients being admitted, transferred, or discharged within four hours [4], when the target is 95%.

Much less publicised and understood are the implications of these challenges for staff delivering care. Most crucially, we understand little of the impact on their emotional experiences and how they manage associated emotions [5, 6].

This matters because emotional health is intrinsic to healthcare workers' wellbeing. Considering workers in emergency care are more likely than other healthcare workers to experience poor wellbeing [7], suffer psychological illness [8], and leave their roles [9], this importance is significant.

### 1.2. Hochschild's Emotional Labour (1983): Application to Nursing

Goffman [10] originally discussed the process of “being public” with emotions. This sociological understanding of emotion works with the idea that individuals consciously manage their emotions to follow the values and beliefs within a specific situation or community [11]. Goffman [10] described “front” and “back” stage—the former requires “face work,” in which individual actors have direct control over their “performance” and the management of their emotional performance [11].

The concept of emotional work, and specifically the theoretical concept of emotional labour, products of Arlie Hochschild's The Managed Heart (1983), offered those studying the workplace a new lens through which to explore workers' emotions [12]. Based on empirical studies of flight attendants and debt collectors, both groups suppressed and limited the emotions that they outwardly displayed to the various audiences [13]. In other words, they managed their emotions in response to their surroundings and audiences.

Employees shape and mould their emotional displays in response to the requirements of their institution or organisation, which is seen to constrain these efforts [12]. Hochschild therefore saw the commodification and control of feeling as an often-exploitative process [14]—feelings are exchanged for a wage. It is important to note that this is different to emotional work. Emotional work also involves the manipulation of emotion but is outside of the workplace, free from organisational expectations and control. Emotional work is the process of managing and then presenting our emotions to our family and friends [15]—the private aspects of our lives outside of the workplace.

Emotional labour, therefore, is “the management of feeling to create a publicly observable facial and bodily display” ([13], p.7). This acting is driven by enculturated feeling rules—standards in feeling management—determining what is acceptable between individuals in “the currency of feeling.” Rules are influenced by managerial structures and control systems, ultimately intending to shape emotional displays which support organisational interests [16].

The emotional labour of various professional groups has been examined and perhaps unsurprisingly has included healthcare workers. It is well established that nurses in particular “do” emotional labour routinely in their practice [5, 6, 17, 18].

Nurses are described as “emotional jugglers,” with the capability to present a spectrum of different and appropriate faces ([11], p.97) to remain “professional.” Nurses “calibrate their performances according to the frame of action, choosing whether to match feeling with face” ([11], p.97). More specifically:

… As a distinctive occupational group nurses are particularly adept at changing faces; seemingly effortlessly moving from cynical to sincere, from backstage to frontstage. They are able fully to embrace certain aspects of their allocated role, whilst distancing themselves from others…

([11], p.98)

Such descriptions give insights into the diverse management of emotions of nursing practice, depicting the nurse as a social actor and performer [11], adapting to meet various needs. These terms align strongly with Goffman's [19] “presentation of self” in which employees adopt a specific outward appearance of the expected emotions for the “stage” while their true emotions remain suppressed and out of sight [11, 20]. The spectrum of emotion managed by nurses is also influenced by the nature of the physical tasks undertaken. By its very nature, nursing work, Menzies [21] argues, includes tasks that by usual standards are (at times) disgusting and frightening, requiring the nurse to perform emotional labour. By suppressing true emotion outwardly, the nurses' acts reassure the patient and allow for closeness or a degree of privacy between the patient and the nurse, ensuring that a trusted and intimate relationship can be formed and maintained. Theodosius ([6], p.33) writes:

… The role of emotional labour in nursing is an essential part of the exchange between the individual being cared for and the carer. Feeling rules based on the ideal of nurses being naturally caring operate as “moral” guidelines by which the patient allows the nurse to care for their intimate physical body, and by which they can impart personal and private information about their feelings, thoughts, and way of life. Such a relationship, usually part of intimate private family life, is reproduced in nursing care and is a crucial component of nurses' emotional labour…

At the same time, it is routine for nurses' work and their subsequent emotional labour to include bereavement, frustration, and happiness [22]. Nurses often support and comfort patients and their relatives in times of suffering, when they are dying and at times of immense happiness (e.g., the birth of a child or receiving life-changing news) [23]. This diverse and sometimes dichotomous catalogue of emotions also distinguishes nurses' emotional labour from that of others. In these intense and highly emotional circumstances [24], it is often the nurse who experiences the most significant impact of the emotions involved [23]. Ultimately, here lies an acknowledgment of the additional and complex form of labour, associated with caring for people who are sick [25]. This labour can be emotionally draining and effortful [20] and, as noted above, is intrinsic to experiences of wellbeing.

### 1.3. “Moderating” Emotional Labour

Theorists[26, 27] have explored factors which shape how, and to what degree, emotional labour is performed. They have identified the “moderators” of emotional labour—physical and nonphysical elements which affect the emotional performance in terms of intensity, frequency, and, therefore, the personal impact of the labour.

The examination of moderators has often focussed on “micro” relational considerations [26], for example, an individual's psychological traits (emotional abilities or values) (e.g., [28, 29]). This dominant perspective shows that the negative implications of emotional labour will be lower for employees who embody organisational values [30].

At a meso, or organisational level, scholars have investigated contextual factors which influence experiences of emotional labour including job status; degree of autonomy in work; and social support available to employees [26]. This resource-based perspective acknowledges that emotion regulation will be less depleting, in terms of worker wellbeing, where more resources are available. For example, the perception of strong organisational support systems helps to mitigate the negative effects of emotional labour and in particular its influence on job satisfaction [31].

This is important as emotional labour is not without “cost” to the worker with well-established links to poor wellbeing (e.g., burnout, stress, and intention to leave employment) [20, 32–34]. Understanding moderators can help in alleviating negative impacts on worker wellbeing. When considering the healthcare workforce crisis in the UK [35] and globally [36, 37], this understanding is crucial.

### 1.4. Research “Problem” Formulation: “Time” as a “Macro” Moderator in ED?

We might assume that “macro” workplace considerations like environmental structures may also moderate employees' experiences of emotional labour. Despite this, a thorough exploration of various structural “macro” elements, and their part in the moderation of emotional labour, is limited. Our first paper [38] from the same study showed how spatial elements of the ED moderated emotional labour.

Research within the interesting and unique case study of the ED is an obvious choice for this exploration. The ED is a strikingly pressurised, physically crowded, and time-critical environment and is, crucially, short of resources, and it is emotionally intense. The workforce are struggling to meet growing patient demand and must manage strictly monitored governmental time-critical targets and a diverse and variable patient population, all while meeting their patients' expectations. It is likely that ED nurses' emotional labour is distinctive and moderated by the environmental structures of the ED.

Despite the highly charged nature of ED, we still understand little of how emotions are managed here and subsequently, how this labour could be supported. From an applied perspective, this is particularly important; ED staff are already increasingly vulnerable to poor wellbeing outcomes when compared to other clinical specialities.

### 1.5. Research Questions

Why and how is emotional labour accomplished in the ED?

Which environmental elements of the ED moderate the emotional labour undertaken?

## 2. Methods

The Consolidated Criteria for Reporting Qualitative Research (COREQ) guidelines have been used for preparing this paper.

### 2.1. Data Collection

This paper focusses on the experiences of a group of emergency nurses at two NHS hospitals in England. We undertook an ethnography of two EDs, one in a Teaching Hospital, including major trauma centre, and a District General Hospital. Data were generated over a 6-month period. In total, 200 hours of observation were completed.

#### 2.1.1. Ethics

All relevant NHS and University ethical approvals were obtained.

#### 2.1.2. Observation

An ethnographic method was the primary source of data collection, and this approach gave space to unpack both hands-on practices and the discrete behaviours [39] associated with the management of emotion.

Internal management and suppression of emotion is subtle, hidden, and often complex in its presentation. The doing of emotional labour by professionals may be, in many cases, so entwined in daily healthcare practice that staff have no conscious awareness of it. Direct observation in the field of the ED offered a means of witnessing the behaviour of healthcare staff directly and therefore an opportunity to see the culture and practice of their emotional labour first-hand.

All areas within the ED (outside the department; the reception area; minor injuries area; initial assessment; majors area; resuscitation; all adjoining corridors; staff-only clinical spaces; and the staff room) were observed both during the day and at night in a 24-hour timeframe. In each area, observation was undertaken from a variety of vantage points, still ensuring that the flow of care was not interrupted. During the periods of observation, countless informal conversations took place between staff (individuals as well as groups) and the researcher.

#### 2.1.3. Interviews

As a means for checking interpretations in the field, 35 semistructured interviews were completed across the two hospital sites. The formal, preplanned interviews focussed on the experiences of ED nursing staff, with varying levels of seniority, all with ED experience and no other demographic criteria. We also interviewed wider support staff of ED including managers and leaders. We used the observational data as a prompt for staff during the interviews, cues from which they could then expand and describe their interpretations and perceptions of their experiences of work. As the observations were looking at the context and the structures around nursing work, the interviews were about the feeling and experiences of working as nurses in this context and how the wider team found emotions to be part of the support they delivered.

The participants were recruited as volunteers via social media and email. We completed the interviews at a location of the participant's choice, both inside and outside of work with no other party present. Interviews were recorded electronically and then transcribed verbatim, which lasted between 30 minutes and 1 hour.

Metaphors can help individuals to communicate the concealed through a well-known and familiar phrase [40] and subsequently can help researchers to study elements of organisational life [41]. Recurrent metaphorical terms used by the ED staff were, therefore, derived from field notes, including “firefighting,” “warzone,” “dungeon” and “bunker”. These metaphors were then used within the semistructured interview topic guide to underpin questionning (see [42]). The nurses used these metaphors as a representation of their working experiences, revealing insights inaccessible without these triggers. Questioning unpacked the understanding and interpretation of the metaphors and their experiences.

### 2.2. Data Processing and Analysis

To protect participant confidentiality, all identifiable information was removed and pseudonyms were assigned. Analysis was interpretive and reflexive, underpinned by “Progressive Focusing” [43]. We also used NVivo software to support the data management process. After an extensive period of familiarisation with the data, initial nodes were derived. These encompassed a mixture of descriptive, analytical and potentially theoretical recurrences. A refined list of nodes and subnodes was then established from each individual source until data saturation was reached. We then worked collectively as a team through coding to a higher level of conceptual abstraction: “time” as a moderator of ED nurses' emotional labour.

### 2.3. Trustworthiness and Processes to Ensure Rigor

Quality judgements in qualitative research are demonstrated through transparency and rigor. We worked collectively as a team to ensure this, challenging subjectivity and its influence upon the subject formation of “social, discursive, and material processes” ([44], p.737). Gherardi ([45], p.23) argues that “plausibility is the validity criterion for ethnographic research.”

Due to the subjectivity of the interpretation process, it is crucial that the researcher outlines all aspects of the methods involved and the data collected [45]. We used ongoing reflectivity to ensure our research processes worked to explore and understand existing bias and preferences and mitigate against them. This was fundamental for our study, particularly when considering the main author's experience as an ED nurse. Reflectivity, therefore, focused on negotiating the strengths and challenges of being both an “insider” and “outsider” to the field. The participants were made aware of the author's nursing background.

Through the collection of data and process of analysis, which started in the field, we took steps to ensure rigor. For example, utilising a combined approach of formal interviews alongside observation offered continuous opportunities for challenging interpretations made in the field, and vice versa. Within the interviews, respondents' recounted experiences were challenged and reaffirmed. Other mechanisms utilised included triangulation; respondent validation; “fair dealing”; and attention to negative cases.

## 3. Findings

… I suppose from the outside it could appear that you're managing well, you're getting to your patients, you're putting on a front, you're smiling, you're happy. You present yourself. Then you're whisking off to take the next patient or moving on to another area. So, yeah… patients' or relatives' perception could be that it doesn't look as busy because they don't see what's going on behind the scenes. They don't see what resus is like, that there's minus two beds in there… Or the walk-in side… there could be probably five or six people in the waiting room wanting to know why they've not been seen straightaway because it doesn't look busy, whereas resus is just behind the doors and there could be massive traumas going off. [Don't let the patients] see you're stressed and flustered and don't have time… It gives them reassurance…

(ED S/N Carly District ED, Formal Interview)

The findings outlined below depict the unique character and ambience of the ED and crucially how various elements of time moderate the emotional labour undertaken. The account which follows focusses on the dimensions of this structural, macro moderator, illustrated through the impact of various time-critical targets. We present the following themes: time-critical targets moderating labour and suppressing stress; “the assembly line”; “zero to hero” transactions and building trust and limiting emotional offerings.”

### 3.1. Time-Critical Targets Moderating Labour

Clinical Services Quality Measures (CSQMs) provide an at-a-glance indication of how well services are “performing.” For EDs, many of the quality indications focus on the length of time needed for specific processes or episodes of care, largely in relation to the following points:

- Left ED before being seen for treatment
- Re-attendance
- Time to initial assessment
- Time to treatment
- Total time in A&E

[46]

Time-critical care delivery is at the heart of emergency care delivery around the world. Arguably, the four-hour wait target is most critical to the way in which care is delivered to patients in English NHS, although similar targets are relevant internationally. It was introduced in response to poor standards of care [47], in which patientswere left waiting in the ED for long periods of time.

The target, described as a Clinical Services Quality Measures (CSQMs) are designed to monitor how well offers an incentive for Trusts to improve waiting times and holds NHS managers to account when the target is not achieved [46]. Trusts are fined when the target is breached without a clinical exceptional justification for the breach. By 2004, the NHS intended that no patient should stay longer than four hours in the ED. Therefore, 100% of patients should be assessed by medical staff; given appropriate treatment or intervention; and then admitted to the hospital, transferred to another speciality, or discharged home [47]. Since then, the target has been changed to lower thresholds, 98% then 95%, and performance in achieving the desired percentage has varied [48]; an all-time low was hit in 2022 with 57% of patients being admitted, transferred, or discharged within four hours [4].

Other quality indicators in ED also focus on the length of time needed for specific processes or episodes of care, largely in relation to the following points:

- Left ED before being seen for treatment
- Re-attendance
- Time to initial assessment
- Time to treatment
- Total time in A&E

[46]

Time to initial assessment on presentation at the ED should be completed within 15 minutes of the patient arriving for all patient groups [49]. Time to initial treatment however will also vary depending on clinical presentation. For example, a patient presenting to ED with chest pain or symptoms of a stroke requires a much quicker time to treatment than many minor injuries.

Feelings of frustration manifest from the ED nurses relating to the maintenance and prioritisation of targets, particularly the four hours. Allowing patients to breach the target, without clinical exception, is seen as unacceptable and can be a source of tension. The tension experiences by the nurses must be hidden, along with a myriad of other emotional experiences. They must undertake their emotional labour to ensure that their patients and colleagues remain unaware of feelings of stress. We found these elements of time to be intrinsic to this emotional labour undertaken.The restrictions on time available to the nurses (a product of various time-critical targets) moderated and intensified the ‘acting' needed by the nurses to ensure their true feeling remained hidden. In the extract below, the nurse suppresses her true emotion and stress as the four-hour clock “ticks” down, restricting their time to manage the patients' care and intensifying the required emotional labour needed to remain “professional”:

Observing the paediatric area, there is anxiety about a patient who is approaching the four-hour breach. He is waiting for a speciality doctor, outside of the ED, to review his X-rays to see what the plan is (whether he be admitted or sent home). The relative, his mother, is becoming more and more agitated, wanting to know what is happening. The ED doctor is on the phone to the speciality doctor. The nurse is dashing between this and the tasks associated with pre-empting the decision–e.g., trying to arrange him a bed, and other clinical tasks for other patients–it is clear that she can't allow the patient to breach the four-hour target and that this is a priority. Because the case has been rushed and involves a last-minute decision, the ED staff are worried about transferring the patient to the ward. This is because of the hostility they are likely to receive from the ward staff.

The tension at the nurses' station is palpable as they await a decision from the speciality doctor and at the same time try to plan for every possible outcome to prevent the breach. A nurse begins to get frustrated, as none of the speciality nursing staff will take the patient's details before the consultant has looked at the X-ray, despite the importance of preventing the breach. The clock is ticking down and the nurse is ringing more-senior staff; the speciality doctor is still unreachable. Frustration and tension are mounting, which can be seen in the nurse's facial expressions and body language. She sighs, has her hand on her hip and looks at her most tense–despite this, her language on the phone has changed very little. She remains calm and becomes a little more assertive but does not raise her voice. With only a few minutes to go, the speciality doctor arrives in the ED. The problem resolves and the patient is admitted to the ward. The release of tension in preventing the breach can be felt profoundly. The nurse explains that most ward staff don't “get it” regarding the priority given to patients breaching the target.

This period of observation showed a clear example of the surface acting required in managing the four-hour target. During the period outlined, the nurse remained outwardly calm (she was not rude, did not raise her voice, and did not change the tone/speed of her speech). However, through her visibly growing frustration, seen in her body language, tension in hands and shoulders, and facial expressions, rolling of eyes and gritting of teeth, it was evident that she was struggling to maintain this exterior as the situation escalated and time was running out. In undertaking this emotional labour, she maintained her calm approach—the colleague on the phone would most likely be unaware of the frustration experienced.

Moreover, after the tension had subsided and the patient had been moved, the nurse reaffirmed to me how she had needed to “bite her tongue.” She also explained how the dynamics of maintaining the four-hour target regularly require this management of emotion. The suppression of feeling, especially frustration, was seen to be an essential component of effective communication, particularly when interacting with those outside of the ED, without the organisational understanding in relation to the target. In this instance, and in the example given above, the ED nurse gives importance and priority to maintaining the target; the ward nurse does not share this priority. In response, the ED nurse undertakes emotional labour to suppress the frustration surrounding the lack of time before the patient breaches the target and there are undesirable repercussions.

### 3.2. The “Assembly Line” and Suppressing Stress

In the UK, as is the case elsewhere, demand outstrips supply in the ED. As such, ambulance crews (often, but not always including paramedics) are unable to hand over the care of their patients to the ED staff and are therefore unable to attend other 999 emergency calls. Ambulance-to-nurse handover should take place within 15 minutes of arrival at the ED in England, and handover times of 30 minutes or more can lead to hospital fines [50]. If the ambulance turnaround time is not met, this affects patient flow through the department, causing a backlog in the ED system, described as “bottlenecking.”

As patients queue along long and thin corridors, the noise of frustrated and irritable patients and ambulance crews is palpable. The state of emotion, angst, and tension builds before patients even arrive at theassessment area. There is constant movement—it is difficult to keep track of the processes:

… never stationary and constantly changing. As soon as one patient's assessment is complete, they are wheeled out and another is wheeled in–rarely are the cubicles empty. There is an “assembly-line” approach to this space. The next ambulance crew are called to bring in their patient as soon as soon as a cubicle comes available. There is no time in-between each case to reflect–before staff have finished writing notes or tasks for a patient, another has been put in their place. The pace is intense, unforgiving and constant.

(Observational Notes, Teaching ED)

In response to the time-critical targets (e.g., organisational and governmental) and time-critical care interventions (e.g., time-critical medications and tests), the interactions between nurse and patient are often fleeting and intense. There is an “assembly line” nature to the interactions and care delivered here—a metaphor used by many staff sharing their experiences of working in this space. The nurses therefore “do” emotional labour to suppress their emotional experiences associated with not having enough time, for example, frustration, stress, and worry, to present outwardly the “face” the patient is expecting and arguably needs. They do this to ensure that the patient remains unaware of the time limitations and nurses' true feelings. ED S/N Bev explains:

I've actually used that term [assembly line]–it's like a production line of patients… you've got [ambulance] crews coming in constantly. You take handover from the crew, do the basics, move on to your next patient. Take handover, do the basics, move on to your next patient. You might not even see that patient again…. it's all about keeping the flow of the department going… it means there's a definite lack of care there, I would say…

This labour is intensified further still by the duration of the shift, often 12 hours with minimal breaks, and periods of constant working productivity. There is little, if any, reprieve from the performance. In addition, the ED environmental layout makes for minimal “off stage” space and constant visibility to colleagues and patients (see [51]).

ED S/N Adam talks about his experience of completing his nursing assessments. He refers to the restrictions on time (through time-critical targets) to complete the required assessment and tasks, but also the length of the shift time cycle, through which the work and subsequent “mask” is maintained:

There is a big time element, which puts a lot of pressure on you because the idea is… Within 15 minutes of the ambulance putting their handbrake on outside, the crew are meant to be in, transferred, handed over and then back out in their vehicle… Then can you imagine on this particular day we had patients coming into the cubicle who'd been booked in for an hour and a half? It's hugely frustrating. When it gets to that stage… relentless… powerless about the situation… you get the pressure from the crew standing there staring at you… “Why aren't you working a little bit faster?” What I'd like to say to them is “please come and try working here for twelve and a half hours.”

(ED S/N Adam, Teaching ED, Formal Interview)

### 3.3. “Zero to Hero” Transactions and Building Trust

The restrictions on time directly influence the nature of the nurses' work, the relationships the nurses develop with their patients, and the necessary emotional labour. Although the nurses have less time with each patient, the patient expectations from the nurse (kindness, patience, calm, etc.) are still present. Arguably they are at their most intense in ED, and patients are likely to be scared on arrival, in pain and suffering.

In response, the ED nurse must go from “zero to hero” (taken from the extract below) during the fleeting and transient interactions. The ability to build a rapport and intimacy with patients in a minimal timeframe is an essential component of ED nursing. It is important that the patient is unaware of the time limitations and pressures felt by the nurse as this would exacerbate their feelings of fear and uncertainty. Rapport must therefore be built at speed, as the nurses often only spend “snippets of time” with each patient. Sue explained:

They [ED nurses] build a relationship with that individual enough to get that part of the job done. 'Cause you've got to go from zero to hero, as far as I'm concerned… Never knowing somebody to doing something really, really intimate… So you've got to get to know them really quickly, for them to be able to trust you.

(Deputy Divisional Nurse Sue, Teaching ED, Formal Interview)

In addition, the minimal time between patients leaves no downtime for the nurses. They must move between two or more cubicles interchangeably and within seconds. These intimate interactions are constrained, pressurised, and intensified by the limited time available. As a result, the nurses must change faces or masks at speed to ensure their emotional exterior meets the need of each patient, suppressing any conflicting emotion such as anxiety, fear, and annoyance, through their emotional labour.

As argued by Kirk et al. [38], the distinctive nature of the ED in terms of patient population means that staff also routinely move between a spectrum of clinical and emotional need, for example, minor injuries and major trauma. This also holds significance for the intensification of emotional labour involved. Patients are often only separated by thin disposable curtains, which leaves seconds for the changing of “multiple masks.” Moving between these patients with little time in between intensified the acting for staff. The case study example below is an illustration:

In one cubicle is a 25-year-old male; he is of a large stature and build. He has taken illegal drugs and is intoxicated after attending a music festival. He has been brought to the ED after collapsing and requires a period of cardiac monitoring. His heart rate is abnormally and dangerously high–a consequence of the drugs consumed. He is awake and conscious, is verbally aggressive towards staff, is pulling off clinical monitoring equipment and wishes to leave the ED, against medical advice. He is there with his girlfriend, who has also been drinking alcohol and is verbally aggressive towards staff.

Directly next to him is an elderly lady who has end-stage cancer and is receiving palliative care. She has been brought to the ED with an infection that cannot be managed in the community setting. She is gravely unwell and despite treatment is not expected to survive; she has a “do not resuscitate” (DNR) order in place. She is waiting for a side room on a medical ward. She is drowsy but polite to staff and is reassured by their communication and company. She has no family locally so attends the ED alone.

There are many instances when the spectrum of need between patients is this notable. The physical time available between these patients exacerbates the emotional labour required to achieve reassurance and empathy for both parties.

(Observational Notes, Teaching ED)

### 3.4. Limiting Emotional Offerings and Managing Guilt

Many of the nurses described how they found restrictions and limitations on their time upsetting and even guilt-inducing. They knew they did not have enough time to give every patient the standard of care the patient needed, or the standard of care the nurse wanted to deliver. As noted above, often emotional labour was used to internalise these feelings and still present the expected front. However, for some nurses, they felt so short on time that they used their emotional labour to restrict emotional offerings to patients. Staff limited themselves during their interactions with patients. In this sense, their acting was used as a defence against connecting with patients and was a form of emotional dissonance. ED S/N Becky offered her own experience:

… it is [the time pressure], makes me consciously avoid asking [patients] certain questions because I think: “Well, if I do that, I open the floodgate,” so I'm probably keeping my interactions with patients and relatives much shorter than I have done previously.

How does that make you feel?

… I don't think that it's very helpful. Putting up a bit of a defence… I know if I ask certain questions or if I go and try to find out certain answers, it's going to take time. I haven't got time to do that. Then that makes me feel bad that I can't do it… where I worked before, a lot of our patients would come in and out [for treatment]. So we would know them. I think I'm having to adjust from having that sort of relationship to just stabilising patients and being able to reassure them but probably not in a very meaningful way, which is what I was able to do before.

(ED S/N Becky, Teaching ED, Formal Interview)

There was often a misalignment of priority for the nurses of achieving the required time-critical targets, limiting emotional connection in response and a desire for connection, intimacy, and a slower pace (arguably driven by more “traditional” nursing values). For some, this disconnect proved unmanageable and staff explained how it impacted upon their wellbeing. In some cases, the nurses described to us how they left their role:

… I think you need to be quite stony-hearted because it's a hard place to work… I care too much. I can't walk past somebody that says: “Can you help me?,” and unfortunately you don't have time. In A&E, you don't have time to stop for every person who says “excuse me.” You need to be able to walk past people… I think it's one of the major things that make it a hard place to work because you feel that you're not doing the best for the people you're looking after… it can actually grind you down. As nurses, you want to care for people. You want to make a difference… you can't always do that in A&E…

(Former ED Nurse Michelle, Teaching ED, Formal Interview)

## 4. Discussion

In this paper, we seek to understand how time moderates the emotional labour required by ED nurses. Here, time refers to specific time cycles, including the proportion of time spent working in periods of productivity [52]. Both the length of shift and frequency of ED nurses' productivity are notable—the amount of nonproductive time in a shift, for example, is often minimal. This intensifies the nature [52] of the ED nurses' work and, notably, the implications for their emotional labour; acting must be sustained for these long periods. Such experiences are amplified further through the lack of “off-stage” space and constant visibility for staff [38].

Arguably, however, the time-critical targets of ED dominate the emotional labour undertaken. These have the most notable influence over the nurses' work because they result in the restriction of time for various nursing care and patient interactions. Noon and Blyton [52] acknowledge the implications of employees feeling that they have “too little time” at work, especially to complete allocated tasks. Work intensification (see [53]) can offer insight into the historical developments of workload, more recently encompassing the emotional and mental component of work (seen as qualitative components) alongside quantitative job demands (the pace and amount of physical work). The consequences of increased work intensification have been established as negative for employees' wellbeing and health [54, 55]. This was certainly the case for the ED nurses and, most crucially, they enacted significant emotional labour to hide the stress and other emotion associated with the intensification of their work.

Because of the work intensification in ED, the lengths of time cycles, and the quantity and complexity of the work that needed completing within them, the interactions between nurse and patient were brief most of the time. Existing literature tells us that the actual duration of emotion display is relevant to how intense the work is deemed to be. Previous research has argued that short interactions require less effort than those that are over a long period. This is because shorter interactions in service roles tend to be scripted [56], requiring less depth of emotional labour. Conversely, in longer interactions, the relationship between employee and customer develops: the employee starts to relate to the customer and subsequently finds it harder to hold in personal feelings and relatability [57]. Moreover, intensity and duration are also related. Long interactions are more likely to require the display of more “intense” emotions. Morris and Fieldman ([57], p.991) write:

Thus, clients do not expect emotional intensity in short scripted interactions with tele-marketers, but they do expect more intense exchanges in longer, non-scripted interactions with nurses…

Based on the findings presented, this paper does not support the arguments highlighted above by Morris and Fieldman [57]. Despite the often brief nurse-patient interactions in the ED, and consequently the short “bursts” of emotional labour, the level of connection expected between a patient and a nurse is still expected to be sincere. Pytel et al. [58] found that a top priority for patients visiting ED was communication with the nurse; they prioritised compassionate communication that showed genuine concern. Uy et al. [59] explain that by prioritising speed and efficiency in service roles, achieving quality service interactions (between customer and employee) becomes more challenging. This is amplified in ED; not only do the interactions need to be short to meet the time-critical targets, but at the same time, the degree of concern felt from the interaction is high. Patients are often at their most stressed and scared in the ED.

We argue that the transient interactions in the ED are intensified by the short duration, not eased, which the existing literature would suggest. These interactions are heightened further when time-critical clinical conditions are involved and a patient is likely to deteriorate as time passes. The lack of “scripted” emotional labour, the element ensuring that short interactions are easier for employees [56], is also relevant. The emotional labour of the ED nurses is the opposite: not scripted and often impromptu or improvised, particularly in response to challenging patients—those who are aggressive in nature, are intoxicated, are under the influence of drugs, or have other communication issues (perhaps they are acutely medically or mentally unwell). This may offer an explanation as to why a short duration of interaction, in this case, does not decrease the level of intensity.

Prior to this research, the sociology of emotions, including the study of emotional labour, has often focussed on microanalysis, between individual social actors, with little attention to the influence of meso and macro structures [60, 61]. Understanding the moderators of emotional labour, namely, time from a meso, organisational perspective, offers further theoretical advancement.

## 5. Recommendations for Practice

The more “sped up” a service is required to be and the more the work is intensified, the higher the likelihood of emotional labour is [13, 59]. In light of the challenges facing healthcare services around the world and the increased throughput through services (particularly in ED), these are important considerations.

This is also critical when considering that there are well-established relationships between emotional labour and wellbeing in nursing. Psychological wellbeing (mental and emotional health) in nursing is associated with poor experiences of work, intention to leave, and poor retention [33, 62, 63]. The psychological wellbeing of UK nurses was amongst the worst in the world prior to the COVID-19 pandemic [64]. In the most recent NHS Staff Survey, over 50% of nurses said they had felt unwell as a result of work stress in the last 12 months and around 60% had come to work in last 3 months despite not feeling well enough to do their duties, known as “presenteeism.” At the same time, there are already a workforce crisis in health and social care in the NHS, with staff shortages and a highly stressed workforce still recovering from the COVID-19 pandemic. The workforce is in crisis: a product of funding shortages, inadequate policy, and prolonged poor workforce planning. By 2030/31, half a million extra healthcare staff are needed to meet rising demand for healthcare—a 40% increase in existing workforce [35].

Sickness absence rates in the NHS are higher than those in the rest of the UK economy [65], and 44.8% of staff felt unwell in the last 12 months as a direct result of workplace stress [66]. NHS sickness represented more than 1.4 million full-time equivalent days lost in April 2019 alone [65]. Recent analysis by IPPO, the University of East Anglia, and RAND Europe estimates that the cost of poor mental health and wellbeing to the NHS might amount to £12.1 billion per year [67]. These trends reflect the workforce crisis globally.

Poor experiences of work and wellbeing in the healthcare workforce affect levels of compassion and professionalism [65]. Healthcare workforce wellbeing poses an enormous threat to patient safety through clinical error, infection rates, and standardised patient mortality figures [63], and staff burnout is associated with medical error [68]. Perhaps unsurprisingly, staff wellbeing shapes not only patient outcomes but also their experiences of care [64, 69].

At a time of crisis in the NHS workforce, with high stress and burnout, the psychological legacy of the pandemic, and poor staff retention, this study is very timely. Understanding the relevance of the healthcare environment to staff members' experiences of emotional labour, therefore, is likely to be a useful tool in counterbalancing the potentially negative consequences of this work. In particular, understanding the relevance of environmental elements, namely, time, to the emotional labour offers tangible opportunities for improvement. Despite this, emotions and wellbeing are often labelled as “soft” and can be easily overlooked compared with more technical aspects. This is limiting the healthcare sector's ability to retain staff and provide high quality patient care.

What can be done in response? The fundamental basics of staff experience such as adequate resourcing should be prioritised [70]. This includes economic investment and long awaited mandated safe staffing ratios to alleviate work intensification. Concurrently, Maben et al. [69] argue that the “individualistic approach” to wellbeing is the “primary strategy” in healthcare management despite being more detrimental for staff psychological emotional wellbeing; placing the emphasis on individual resilience as an inherent quality is damaging to staff and ignores organisational responsibility [71]. This is shift is of particular importance in clinical areas such as the ED where work intensification is on an upward trajectory and professional identity drives the need for stoicism and unwavering personal resilience [38].

Instead, system-wide approaches to managing and discussing wellbeing are essential. A shift to organisational and system level solutions are urgently required [70] and shown to be most effective [72]. The “Courage of Compassion: Supporting nurses and midwives to deliver high quality care” report [65] offers an example of a system-wide approach. The report includes recommendations for how organisations can meet the underpinning core needs of nurses and midwifes for better experiences of work (and workforce wellbeing) through system/organisational change; it highlights three core work needs that must be met to minimise workplace stress: autonomy, belonging, and contribution, and eight accompanying recommendations relating to influence; justice; work conditions and schedules; teamworking; culture; workload; management; and development.

In partnership with adequate resourcing, staffing, and other basic components of safe working environments, Greenberg and Tracy [73] call for evidence-based psychological interventions delivered which take a systems level approach, such as Schwartz Rounds and various forms of supervision. These interventions can help to normalise the emotional responses of contemporary healthcare and help staff work through the emotional complexity of practice. It is important to emphasise, however, that these mechanisms should not be used as standalone “solutions” but in partnership with elementary mechanisms to improve staff's experience of work. Maben et al. [74] found Schwartz Rounds in particular to reduce poor psychological health, reduce isolation for staff, and increase empathy [74]. However, and perhaps ironically, structural and organisational challenges and culture often prevent staff, particularly nursing staff, from attending Schwartz Rounds and affect their ability to access other support mechanisms—again emphasising the importance of initiatives which are part of a whole systems approach. Many of the nurses in our study welcomed any opportunity for “headspace,” reprieve, and genuinely supportive conversations where they could normalise experiences and “check in” with peers about especially challenging cases.

Ultimately, w need a fundamental shift in the narrative related to wellbeing,with the importance of investment for service sustainability and patient safety and improving the health and care experiences of patients and the wider public emphasised. Although the English NHS was used in our study, the findings are applicable internationally. Although specific targets will vary around the world, the time-critical nature of the work in ED is universal.

This research is of relevance to all clinicians of the ED, but of particular importance to nurses due to the nature of their work and patient contact. Other health services where increased demand is impacting upon the time available for patient interaction can also draw insight from this research.

## 6. Limitations

As with all ethnographic studies, direct application to other settings [75] should be taken with caution. However, it is anticipated that findings will still be of applied and theoretical value to clinical services in healthcare outside of the immediate ED.

## Data Availability

The data used to support the findings of this study are available on request from the corresponding author. The data are not publicly available due to containing information that could compromise the privacy of research participants.

## Additional Points

*Impact. What Problem Did the Study Address?* (i) Emotional labour: the suppression and management of emotion is central to nursing and critical for the nurse/patient relationship. (ii) Established links between emotional labour and wellbeing, including intention to leave, burnout, and compassion fatigue. (iii) Yet we understand little of how the environment moderates emotional labour, and our study addressed this problem in the ED. (iv) New understandings can underpin solutions to negative consequences of this work. *What Were the Main Findings?* (i) We have shown new insights into nurses' experiences of emotional labour in the emergency department (ED). (ii) Our paper conceptualises “time” as a “macro” moderator of this emotional labour, and the time-critical targets of ED dominate the emotional labour undertaken. (iii) The short duration of interactions in ED intensifies emotional labour, not easing it (which existing literature argues). *Where and on Whom Will the Research Have an Impact?* (i) Less damaging emotional labour means better staff wellbeing, better staff wellbeing means better retention, and better retention means better patient care. (ii) Practically, this research and future subsequent research will impact nursing managers, front-line staff, policy makers, and those tasked with designing and leading services. (iii) This research also makes an important contribution/impact theoretically, filling a gap in existing knowledge relating to the moderators of emotional labour. *Reporting Method*. The Consolidated Criteria for Reporting Qualitative Research (COREQ) guidelines have been used for undertaking and preparing this paper. *What Does This Paper Contribute to the Wider Global Clinical Community?* Although the English National Health Service has been used as a case study for this research, the contributions and impact are internationally relevant. Globally, the challenges facing the healthcare workforce, public-funded health systems, and EDs are shared. Emotional labour is universally relevant offering a significant contribution to the global clinical community.
